# A PQ-loop protein Ypq2 is involved in the exchange of arginine and histidine across the vacuolar membrane of *Saccharomyces cerevisiae*

**DOI:** 10.1038/s41598-019-51531-z

**Published:** 2019-10-21

**Authors:** Miyuki Kawano-Kawada, Kunio Manabe, Haruka Ichimura, Takumi Kimura, Yuki Harada, Koichi Ikeda, Shiho Tanaka, Yoshimi Kakinuma, Takayuki Sekito

**Affiliations:** 1 Department of Bioscience, Graduate school of Agriculture, Ehime University, 3-5-7 Tarumi, Matsuyama, Ehime 790-8566 Japan; 2 Advanced Research Support Center (ADRES), Ehime University, Matsuyama, Ehime 790-8566 Japan

**Keywords:** Biochemistry, Cell biology, Molecular biology

## Abstract

In nutrient-rich conditions, basic amino acids are actively accumulated into the vacuoles by H^+^-coupled transporters in *Saccharomyces cerevisiae*. In addition to the H^+^-coupled systems, the existence of an exchanger for arginine and histidine was indicated by kinetic analysis using isolated vacuolar membrane vesicles; however, the gene(s) involved in the activity has not been identified. Here, we show that the uptake activity of arginine driven by an artificially imposed histidine gradient decreased significantly by the disruption of the gene encoding vacuolar PQ-loop protein Ypq2, but not by those of Ypq1 and Ypq3. The exchange activity was restored by the expression of *YPQ2*. Furthermore, the substitution of a conserved proline residue, Pro29, in Ypq2 greatly decreased the exchange activity. These results suggest that Ypq2 is responsible for the exchange activity of arginine and histidine across the vacuolar membrane, and the conserved proline residue in the PQ-loop motif is required for the activity.

## Introduction

The vacuole in yeasts serves as a storage compartment for amino acids^1^. In nutrient-rich conditions, approximately 50% of cellular free amino acids are distributed within the vacuole, and the majority of basic amino acids, such as lysine, histidine, and arginine, are particularly accumulated in this organelle. The vacuolar amount of basic amino acids is severely reduced in *vma1*Δ cells defective in the vacuolar type H^+^-ATPase (V-ATPase)^2^, which suggests that V-ATPase plays a crucial role in the vacuolar compartmentalization of these amino acids. In a kinetic analysis using isolated vacuolar membrane vesicles, it was indicated that 10 amino acids (arginine, lysine, histidine, phenylalanine, tryptophan, tyrosine, glutamine, asparagine, isoleucine, and leucine) are taken up actively into vacuolar membrane vesicles prepared from the wild-type strain of *Saccharomyces cerevisiae*^3^. Many of genes involved in the uptake activities have been currently identified by reverse genetics and investigated using *in vitro* transport experiments with vacuolar membrane vesicles, and their uptake activities have been suggested to be H^+^-coupled systems^4–6^.

Recently, we indicated that Ypq1, Ypq2, Ypq3, and Avt1 are major determinants for the vacuolar uptake of basic amino acids, based on the transport assays using isolated vacuolar membrane vesicles^7^. Among these proteins, Avt1, a member of the *AVT* family transporters that belong to the Amino Acid/Auxin Permease (AAAP) superfamily, is involved in the uptake of neutral amino acids and histidine^8^. Ypq1, Ypq2, and Ypq3 are members of PQ-loop proteins in the lysosomal cystine transporter (LCT) family that belongs to the Transporter-Opsin-G protein coupled receptor (TOG) superfamily^9,10^. PQ-loop proteins possess two well-conserved repeat sequences termed PQ-loop motif, including a characteristic proline-glutamine dipeptide^11^. The mammalian PQ-loop protein PQLC2 and nematode one LAAT-1 are suggested to function in the transport of basic amino acids across lysosomal membranes^12,13^. Cystinosin, another mammalian PQ-loop protein that is distantly related to Ypq proteins, mediates H^+^-coupled cystine export from lysosomes^14^. Both Ypq1 and Ypq2 are indicated to be involved in the uptake of arginine, because the uptake of arginine was partially impaired by the disruption of *YPQ1* or *YPQ2*^7,9^. In certain conditions, Ypq3 may also contribute to the uptake of histidine, because histidine was significantly taken up by the vesicles overexpressing Ypq3^7^.

In addition to H^+^-coupled systems, kinetic studies have pointed out the existence of a high affinity arginine uptake system by exchange mechanism with histidine^15^; however, the molecule(s) responsible for the activity remained unexplained so far. To find a clue as to the gene(s) participating in the vacuolar exchange of arginine and histidine, we investigated the exchange activity of amino acids of mutant *ypq1*Δ, *ypq2*Δ, *ypq3*Δ, or *avt1*Δ. In this study, we revealed the involvement of *YPQ2* in the exchange of amino acids using transport assay with isolated vacuolar membrane vesicles. The role of the PQ-loop motif in yeast PQ-loop protein Ypq2 is also discussed.

## Results

### Histidine-stimulated arginine uptake activity was decreased by the disruption of *YPQ2* or *AVT1*

**Figure 1:**
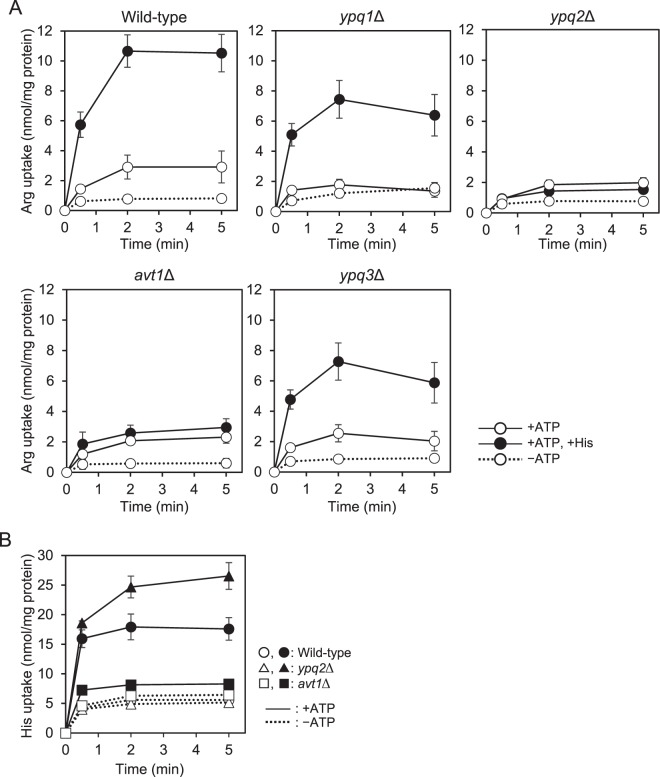
Effect of histidine on the ATP-dependent arginine uptake by isolated vacuolar membrane vesicles. (**A**) Vacuolar membrane vesicles isolated from wild-type, *avt1*∆, *ypq1*∆, *ypq2*∆, or *ypq3*∆ cells were preincubated with (solid lines) or without (dotted line) 0.5 mM ATP for 1 min at 25 °C. The reaction was initiated by the addition of 50 μM [^14^C]arginine at 0 min. 500 μM histidine was added 1 min before to start the reaction (black circles). Results are indicated as mean ± SD from 4~6 independent experiments. (**B**) Vacuolar membrane vesicles isolated from wild-type (circles), *avt1*∆ (squares), or *ypq2*∆ (triangles) cells were preincubated with (solid lines) or without (dotted line) 0.5 mM ATP for 1 min at 25 °C and then 500 μM [^14^C]histidine was added. After further incubation for 1 min at 25 °C, 50 µM non-laneled arginine was added at 0 min. Results are indicated as mean ± SD from three independent experiments.

Recently, we suggested that Avt1, Ypq1, Ypq2, and Ypq3 are mainly involved in the uptake of basic amino acids into isolated vacuolar membrane vesicles, because vesicles of a quadruple mutant *avt1*Δ*ypq1*Δ*ypq2*Δ*ypq3*Δ showed only basal uptake activity for them^7^. In a previous study, exchange activity of arginine and histidine was discovered by analyzing the enhancive effect of histidine on the ATP-dependent uptake activity with a low concentration of arginine as the substrate^15^. Thus, we first examined the effect of disruption of *AVT1*, *YPQ1*, *YPQ2*, or *YPQ3* genes on the exchange activity of arginine and histidine. The uptake activity of 50 µM of [^14^C]arginine by vacuolar membrane vesicles of wild-type cells was strongly enhanced in the presence of 500 µM of histidine, as indicated previously^15^ (Fig. 1A). The enhancement of arginine uptake activity was also observed in vesicles of *ypq1*∆ cells. However, the enhancement of arginine uptake in the presence of histidine was not totally observed and decreased strongly in vesicles isolated from *ypq2*Δ and *avt1*Δ cells, respectively. Uptake of the added [^14^C]histidine (500 µM) in the presence of 50 µM arginine into wild-type, *ypq2*∆ and *avt1*∆ vesicles was also examined. Under the experimental condition, histidine was taken up in a manner dependent on ATP into the vesicles isolated from wild-type and *ypq2*∆ cells, whereas histidine uptake in the presence of ATP into *avt1*∆ vesicles was largely decreased (Fig. 1B). Although Ypq3 was also suggested to participate in the ATP-dependent uptake of histidine into vacuoles, like as Avt1^7,8^, disruption of *YPQ3* had little effect on the activity (Fig. 1A). Therefore, accumulation of histidine in the vesicles under the experimental condition was suggested to mainly depend on *AVT1*.

### Arginine uptake coupled with downhill movement of histidine was dependent on *YPQ2*

**Figure 2:**
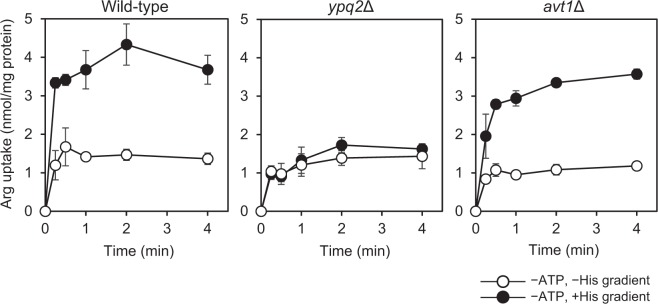
Arginine uptake driven by an imposed histidine gradient in the absence of ATP. Vacuolar membrane vesicles containing 10 mM histidine were prepared from wild-type, *ypq2*∆, or *avt1*∆ cells. Reactions were started by dilution of the mixture 50-fold with assay buffer containing 50 μM [^14^C]arginine (black circles) or buffer containing 10 mM histidine and 50 μM [^14^C]arginine (white circles). The reactions were carried out at 15 °C. Results are indicated as mean ± SD from three independent experiments.

**Figure 3:**
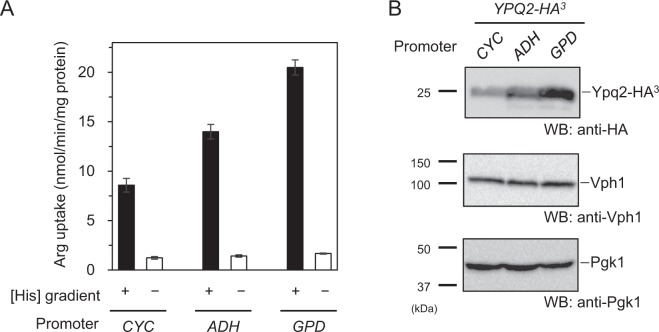
Arginine/histidine exchange activity dependent on the expression levels of *YPQ2*. (**A**) Initial rates of arginine uptake. Vacuolar membrane vesicles containing 10 mM histidine were prepared from the *ypq2*Δ cells harboring pCYC-YPQ2, pADH-YPQ2, or pGPD-YPQ2. The reactions were started by 50-fold dilution of the mixture with assay buffer containing 50 μM [^14^C]arginine (black bars) or buffer containing 10 mM histidine and 50 μM [^14^C]arginine (white bars). The initial rates of arginine uptake were calculated from the data at 30 sec. The reactions were carried out at 15 °C. Results are indicated as mean ± SD from three independent experiments. (**B**) Western blotting analysis of cell lysate. Cell lysates (30 μg of protein) prepared from *ypq2*Δ cells harboring pCYC-YPQ2, pADH-YPQ2, or pGPD-YPQ2 were subjected to SDS-PAGE followed by Western blotting analysis using anti-HA, anti-Vph1, or anti-Pgk1 antibodies.

The volume of vacuolar membrane vesicle was estimated as 5.2 µl/mg protein^16^. Based on the results of Fig. 1B, histidine concentration inside the wild-type vesicles in the presence of ATP was calculated to be approximately 3.5 mM, which was 7-fold of that outside of vesicles, which consistent with the results in the previous studies^17^. Next we examined arginine uptake driven by an artificially imposed histidine gradient in the absence of ATP^15^. Vesicles containing 10 mM histidine were prepared from wild-type, *ypq2*Δ, and *avt1*Δ cells and then diluted 50-fold so as to impose an inside-high histidine concentration gradient at 15 °C, as described previously^15^. The uptake of arginine was observed in wild-type vesicles accompanied by the downhill movement of histidine preloaded in the vesicles (Fig. 2, wild-type) and suppressed in conditions without a histidine gradient. Little uptake was observed in vesicles of *ypq2*Δ cells even though a histidine gradient was imposed (Fig. 2, *ypq2*∆). On the other hand, arginine was taken up into the vesicles of *avt1*Δ cells in the presence of a pre-established histidine concentration gradient (Fig. 2, *avt1*∆), albeit the enhancement of arginine uptake in the presence of histidine was not observed like as in *ypq2*∆ vesicles (Fig. 1A). These results indicate that the *YPQ2* is essential for the arginine uptake driven by the artificially imposed histidine gradient. In addition, the ATP-dependent histidine uptake into vesicles by Avt1 was suggested to be prerequisite for the enhancement of arginine uptake by histidine. When the arginine uptake activities driven by histidine concentration gradient were determined using vesicles of cells expressing HA-tagged *YPQ2* under the control of different constitutive promoters, the initial rates of uptake were increased depending upon the strength of the promoter (Fig. 3A), accompanied with the amount of HA-tagged Ypq2 in the cells (Fig. 3B). Arginine uptake activity was also dependent on the magnitude of the imposed histidine gradient (see Supplementary Fig. S1). Taken together with the results shown in Figs 1 and 2, the exchange of arginine and histidine across the vacuolar membrane was suggested to require the expression of *YPQ2* and a histidine concentration gradient established by Avt1.

**Figure 4:**
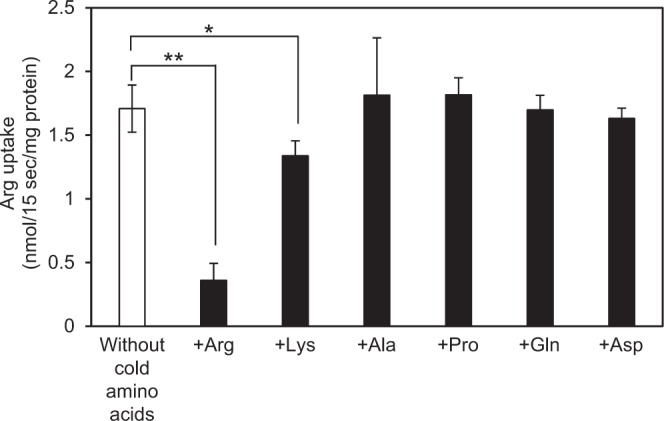
Inhibition of *YPQ2*-dependent arginine/histidine exchange activity by the addition of non-labeled amino acids. Vacuolar membrane vesicles containing 10 mM histidine prepared from wild-type cells were incubated with 50 μM [^14^C]arginine in the presence of 500 μM non-labeled amino acids as indicated (black bars). The initial rates of arginine uptake were determined at 15 sec. The control (without the addition of non-labeled amino acids) is shown as a white bar. Results are indicated as mean ± SD from three independent experiments. Significant differences from the control are indicated by asterisks (**p < 0.01, *p < 0.05, Student’s *t*-test).

To determine substrate specificity of the exchange activity, we examined the effect of various amino acids on *YPQ2*-dependent exchange of arginine and histidine (Fig. 4). The histidine concentration gradient-dependent uptake of [^14^C]arginine was severely inhibited by the addition of a 10-fold concentration of non-labeled arginine. The addition of lysine slightly but significantly inhibited the uptake of arginine, suggesting that Ypq2 could recognize lysine as its substrate, although with lower affinity than arginine. Other neutral or acidic amino acids, such as alanine, proline, glutamine, or aspartic acid, did not affect the exchange activity.

### Substitution of a proline residue in the PQ-loop motif of Ypq2 decreased the uptake activity of arginine

**Figure 5:**
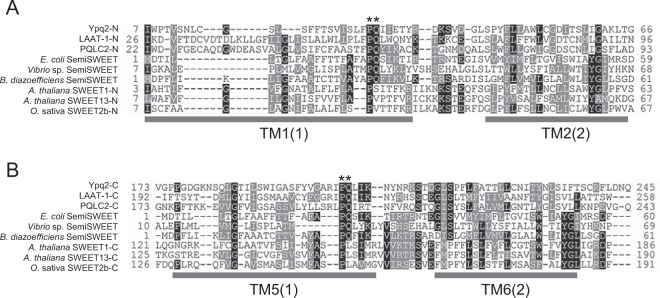
Sequence alignment of the LCT family, SemiSWEET, and SWEET proteins. The amino acid sequence of Ypq2 and its rat and nematode homologs (PQLC2; AAI58608.1 and LAAT-1; NP_493686.2), SWEET species (SWEET1; OAP18988.1 and SWEET13; Q9FGQ2.1 of *A. thaliana*, SWEET2b; B8A833.1 of *O. sativa*) at each N-terminal side (TM1-2: **A**) and C-terminal side (TM5-6: **B**) and SemiSWEET (*Escherichia coli*; GCI09407.1, *Bradyrhizobium diazoefficiens*; Q89G85.1, *Vibrio* sp. N418; WP_009733724.1) were aligned using ClustalW. The TMs based on the crystal structure of *O. sativa* SWEET2b are indicated as gray bars below the alignment. The numbers of TMs corresponding to SemiSWEETs are given in parentheses. The conserved Pro and Gln residues are indicated by asterisks.

**Figure 6:**
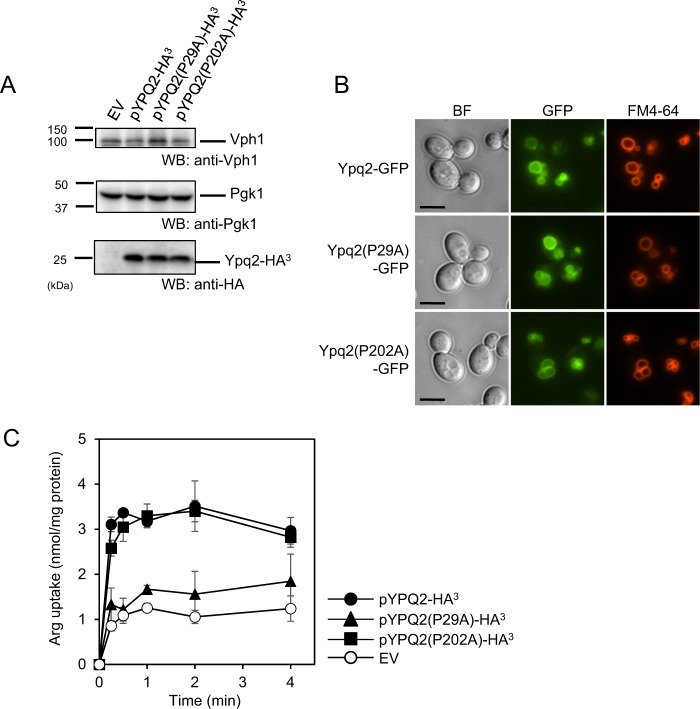
Effect of the Ypq2(P29A) or Ypq2(P202A) mutation on the exchange activity. (**A**) Western blotting analysis of cell lysates. Cell lysates (30 μg of protein) prepared from the *ypq2*Δ cells harboring empty vector (EV), pYPQ2-HA^3^, pYPQ2(P29A)-HA^3^, or pYPQ2(P202A)-HA^3^ were subjected to SDS-PAGE followed by Western blotting analysis using anti-Vph1, anti-Pgk1, or anti-HA antibodies. (**B**) Subcellular localization of GFP-fused Ypq2. The *ypq2*Δ cells expressing either *YPQ2-GFP*, *YPQ2(P29A)-GFP*, or *YPQ2(P202A)-GFP* under own promoter were observed by fluorescence microscopy. The vacuolar membranes were stained with FM4-64. Scale Bar, 5 μm. BF, bright field. (**C**) The exchange activity of arginine and histidine by the vesicles of *ypq2*Δ cells harboring empty vector (EV, white circles), pYPQ2-HA^3^ (black circles), pYPQ2(P29A)-HA^3^ (triangles), or pYPQ2(P202A)-HA^3^ (squares) was determined as described in the legend of Fig. 2. Results are indicated as mean ± SD from three independent experiments.

Although LCT family proteins are distantly related to the plant sugar transporter SWEET (SLC50) in the TOG superfamily, both of them usually have 7 transmembrane helices (TMs) in a well-conserved 3 + 1 + 3 topology^10^. A bacterial SWEET homolog SemiSWEET, which has only 3TMs, was indicated to function as homodimer which results in a similar topological conformation to SWEETs^18^. The PQ-loop motif containing a Pro-Gln dipeptide is well conserved among the LCT family and SemiSWEET, whereas only Pro residues are conserved at the equivalent position in SWEET proteins (Fig. 5). The Pro residue in the first PQ-loop motif was indicated to be essential for the activity of LCT family proteins, because the replacement of the residue with Leu abolished the transport activity of LAAT-1 and PQLC2, respectively^13^. To investigate the function of PQ-loop motif in Ypq2, the Pro29 or Pro202 residue in the PQ-loop motif was substituted with alanine, and the effect on transport activity was examined. Substitution of the residues with alanine did not affect the expression levels of Ypq2-HA^3^ (Fig. 6A). The localization of Ypq2-GFP was not affected by the substitution of Pro residues (Fig. 6B). The exchange activity of the vesicles was severely decreased by the alanine substitution of Pro29 (P29A) (Fig. 6C), indicating that this residue is essential for the exchange activity of Ypq2. In contrast, the alanine substitution of Pro202 (P202A) did not largely affect the activity.

**Figure 7:**
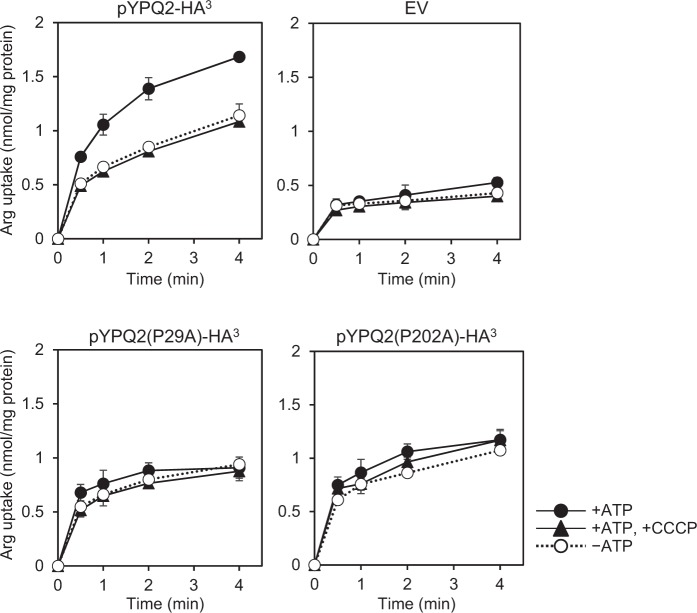
Effect of protonophore on the ATP-dependent arginine uptake activity by Ypq2. Vacuolar membrane vesicles isolated from the *ypq1*Δ*ypq2*Δ cells harboring empty vector (EV), pYPQ2-HA^3^, pYPQ2(P29A)-HA^3^, or pYPQ2(P202A)-HA^3^ were preincubated with (solid lines) or without (dotted lines) 0.5 mM ATP for 1 min. Before initiating the reaction, vesicles were incubated with 5 μM of CCCP at 25 °C for 3 min (black triangles). As controls, vesicles were incubated with solvent (black and white circles). The reaction was initiated by the addition of 50 μM [^14^C]arginine at 0 min. Results are indicated as mean ± SD from three independent experiments.

It has been suggested that *YPQ2* is also involved in H^+^-coupled arginine uptake, because the ATP-dependent uptake activity of arginine was partially decreased by the disruption of *YPQ2*^7^. As described in Fig. 7, ATP-dependent uptake activity was completely abolished in vesicles of *ypq1*Δ*ypq2*Δ cells (Fig. 7, EV), which suggests that both *YPQ1* and *YPQ2* are required for this activity in a redundant manner. Although the activity was much lower than vesicles from *ypq1*∆*ypq2*∆ expressing *YPQ1* (Supplementary Fig. S2), the significant arginine uptake was detected with vesicles of *ypq1*Δ*ypq2*Δ cells expressing *YPQ2*, which was strongly inhibited by the addition of a protonophore carbonylcyanide *m*-chlorophenylhydrazone (CCCP), which dissipates proton concentration gradient generated by the action of vacuolar H^+^-ATPase. This confirmed that the *YPQ2* is involved in H^+^-coupled arginine uptake. The ATP-dependent arginine uptake activity of the vesicles was strongly decreased in the vesicles of cells expressing either *YPQ2(P29A)* or *YPQ2(P202A)*. These results, together with Fig. 6C, suggested that the Pro29 residue in the first PQ-loop motif of Ypq2 plays an important role both in the arginine-histidine exchange and H^+^-coupled arginine uptake, whereas the Pro202 residue in the second PQ-loop motif is required for H^+^-coupled arginine uptake but dispensable for the arginine-histidine exchange.

## Discussion

Based on *in vitro* assays using isolated vacuolar membrane vesicles, it has been suggested that there are three H^+^/amino acid antiport systems for vacuolar uptake of basic amino acids: H^+^/arginine, H^+^/arginine or lysine, and H^+^/histidine uptake systems^3^. The exchange activity for arginine and histidine has also been indicated^15^, although the gene(s) responsible for this activity and its physiological significance have remained unknown. In this study, we investigated the exchange activity of arginine and histidine using isolated vacuolar membrane vesicles and found that this activity is dependent on *YPQ2*.

For arginine uptake coupled with H^+^, it has been indicated that one system is specific for lysine and arginine, because ATP-dependent uptake of lysine was inhibited competitively by the addition of arginine with a *K*_i_ value of 1.5 mM^3^. Since the uptake of lysine was severely impaired and that of arginine was partially decreased by the disruption of *YPQ1*^9^, it was suggested that the uptake activity by the H^+^/lysine or arginine system was dependent on the expression of *YPQ1*. The ATP-dependent uptake of arginine was diminished by the disruption both *YPQ1* and *YPQ2* and was partially restored by the expression of *YPQ2* in *ypq1*Δ*ypq2*Δ cells (Fig. 7). Thus, the other uptake system for arginine with a *K*_t_ value of 0.6 mM^3^ was considered to be dependent on *YPQ2*. It is, however, not clear how the two modes of arginine uptake, of which one is exchange with histidine and another is dependent on the proton concentration gradient, are carried out in Ypq2. In contrast to vesicles of *ypq2*∆, those of *ypq1*∆ exhibited the histidine stimulation of arginine uptake (Fig. 1). In addition, the proton gradient-dependent uptake of arginine in vesicles isolated from *ypq1*∆*ypq2*∆ cells expressing *YPQ1* was much higher than the case that *YPQ2* was expressed in *ypq1*∆*ypq2*∆ cells (Supplementary Fig. S2 and Fig. 7). Therefore, Ypq1 seems to be more specified to the proton gradient-dependent uptake of arginine. Since a more purified system is preferable to investigate its catalytic feature, purification of Ypq1 and Ypq2 so as to reconstitute it in proteoliposomes is ongoing.

Histidine uptake system was inferred to be independent of lysine and arginine^3^. We indicated that Ypq3 is involved in the vacuolar uptake of histidine, in addition to Avt1^7,8^. As shown in Fig. 1, the vacuolar membrane vesicles of *avt1*Δ cells did not show an enhancement of arginine uptake in the presence of histidine, whereas the effect of histidine was observed in vesicles of *ypq3*Δ cells. The cellular level of Ypq3 was indicated to respond for the extracellular concentration of histidine^7^, and to be relatively low in nutrient-rich conditions, which was for the preparation of vacuolar membrane vesicles in this and previous studies^3,7^. Consistently, previous studies indicated that the proton electrochemical gradient-dependent uptake of histidine was mediated mainly by Avt1, and contribution of Ypq3 was only minor^7^. Arginine uptake activity driven by the histidine gradient (inside-high concentration) indicated that vesicles of *avt1*Δ cells accumulated arginine to almost the same level as that of wild-type cells (Fig. 2). These results suggested that the accumulation of histidine in the vacuolar lumen by the action of Avt1 is mainly required for arginine uptake by Ypq2 in the experimental conditions.

It was shown that [^14^C]arginine uptake driven by an imposed histidine gradient was slightly inhibited by the addition of a 10-fold concentration of lysine (Fig. 4). The activity was further suppressed by the addition of a 20-fold concentration of lysine (Supplementary Fig. S3). The addition of a 20-fold concentration of other amino acids, such as alanine, proline, and aspartic acid, did not affect the exchange activity. Therefore, it was suggested that the uptake activity of Ypq2 by the exchange mechanism presumably prefers arginine as its substrate. It is possible that arginine is actively imported and pooled in the vacuolar lumen as a nitrogen source in *S. cerevisiae* by consuming the histidine gradient established by the action of Avt1 and/or Ypq3. In addition, arginine can be also taken up in a manner dependent on proton concentration gradient via Ypq1 and Ypq2. It was, however, implicated that arginine is rather exported from vacuoles via Ypq2, since *ypq2*∆ cells exhibited resistance against canavanine, a toxic arginine analog^12^. The mammalian and *Caenorhabditis elegans* homologs of Ypq2, PQLC2 and LAAT-1, respectively, are suggested to be an exporter of amino acids from lysosomes using cRNA-injected *Xenopus* oocytes^12,13^. In either case, it is plausible that the deletion of *YPQ2* results in the severe shortage of arginine in the cytosol under arginine-depleted condition. To address this possibility, a mutant of arginosuccinate synthetase in the arginine biosynthesis pathway (*arg1*∆) was constructed to avoid *de novo* synthesis of arginine in cells. We assessed the viability of *arg1*∆ cells disrupted with *YPQ1* and/or *YPQ2* in arginine-deprived medium. These cells, however, did not clearly reduced the viability when compared with *arg1*∆ cells (Supplementary Fig. S4). It can be also thought that the vacuolar arginine uptake is to cope with some toxic effects of excess arginine in the cytosol. The growth ability of *ypq1*∆, *ypq2*∆, or *ypq1*Δ*ypq2*Δ cells in the presence of excess arginine were almost the same as those of wild-type cells (Supplementary Fig. S5). Thus, we constructed an arginase mutant cell (*car1*Δ) that cannot catabolize arginine to ornithine and urea. The *car1*Δ cells slightly decreased the growth in the medium containing 500 mM arginine, but the additional disruption of *YPQ* gene(s) did not affect the growth of *car1*Δ cells (Supplementary Fig. S5). Thus, as-yet-unidentified transporter may act in a redundant manner to maintain the growth and viability in these conditions. Identification of such transporter(s) and further study based on the catalytic features of Ypq2 as a vacuolar exchanger of arginine and histidine is required for characterizing the physiological significance of amino acid exchanger(s) operating across the vacuolar membrane in yeast.

To date, the crystal structures of eukaryotic SWEETs and their bacterial homolog SemiSWEETs have been resolved^18,19^. In eukaryotic SWEET, the conserved Pro residues are suggested to form a proline tetrad, and participate in the transport process by a facilitated-diffusion mechanism^19^. The PQ-loop motifs are conserved in the first TM of SemiSWEETs and the first and fifth TMs of LCT family proteins (Fig. 5). In SemiSWEET proteins, the Pro residue in the PQ-loop motif was suggested to function as a hinge for the conformational change associated with the transport process^18^. In LCT family transporters, the Pro residue in the first PQ-loop motif was suggested to be crucial for the activity of PQLC2 and LAAT-1^13^; however, the molecular role of the residue remains unknown. In this study, we found that alanine substitution of Pro29 in Ypq2 severely decreased both histidine and proton gradient-dependent arginine uptake. In contrast, alanine substitution of Pro202 did not affect the arginine-histidine exchange, whereas it decreased the proton gradient-dependent arginine uptake to the same extent as the Pro29 substitution (Figs 6 and 7). This suggested that the Pro29 residue is essential for the transport of substrates by Ypq2 in both transport processes using distinct driving forces. On the other hand, the distinct dependency of the two modes of arginine uptake on the Pro202 residue is implicative of the specific mechanism in Ypq2 for the transport driven by each different driving force. The molecular role of the PQ-loop motif in the transport processes of LCT family proteins should be investigated further.

## Materials and Methods

### Strains and media

*S. cerevisiae* strains used in this study are listed in Supplementary Table S1. For gene disruption of *YPQ2*, the open reading frame (ORF) of *YPQ2* was replaced with a *kanMX* or *natMX* cassette using a PCR-mediated method^20^. Cells were grown aerobically at 30 °C in YPD (1% yeast extract, 2% polypeptone, and 2% glucose) or YNBD + CA medium (0.17% yeast nitrogen base without amino acids and ammonium sulfate, 0.5% ammonium sulfate, 0.5% casamino acids, 20 mg/L tryptophan, and 2% glucose).

### Construction of plasmids

The primers used in this study are listed in Supplementary Table S2. The *YPQ2* ORF with the promoter and 3′ untranslated region, 759 bp and 439 bp, respectively, was amplified by PCR using primers 1F and 2R with *Bgl*II and *Xho*I sites. The PCR products were digested with *Bgl*II and *Xho*I and cloned into a *Bam*HI and *Xho*I-digested pRS316 vector^21^. To construct the *YPQ2* expression plasmid with an HA^3^ or GFP tag (pYPQ2-HA^3^ or pYPQ2-GFP), a *Bam*HI site was introduced before the stop codon of *YPQ2* using a QuikChange mutagenesis PCR and primers 3F and 4R, and a *Bgl*II-digested *HA*^3^ or *Bam*HI-digested *GFP* cassette was inserted. The *YPQ2-HA*^3^ fragment with the 3′ untranslated region was amplified with primers 5 F and 6 R, and cloned into *BamH*I- and *Xho*I-digested p416CYC, p416ADH, or p416GPD, to construct pCYC-YPQ2-HA^3^, pADH-YPQ2-HA^3^, and pGPD-YPQ2-HA^3^, respectively. The alanine substitution of Pro29 or Pro202 residues were performed by PCR-mediated method as above with primers 7F and 8R for Pro29, 9F and 10R for Pro202, respectively.

### Isolation of the vacuolar membrane vesicles

The right-side out vacuolar membrane vesicles of *S. cerevisiae* cells were isolated as described previously^16,17^. In brief, approximately 3 × 10^3^ optical density (OD) of exponentially growing cells were collected and resuspended in 1 M sorbitol containing 200 μg/mL Zymolyase, and incubate at 30 °C for 60 min with gently shaking to make them spheroplasts. The spheroplasts collected by centrifugation were suspended in 6 volumes of Buffer A (10 mM MES-Tris, pH6.9, 0.1 mM MgCl_2_, 12% Ficoll) and homogenized using a loosely fitting Dounce Homogenzer to disrupt cell. The 20 mL of disrupted cells were gently layered on top by 10 mL of Buffer A in ultracentrifuge tubes, and then centrifuged at 50,000 × *g* for 60 min at 4 °C using swing rotor. After centrifuge, the white layer floating on top were collected as the intact vacuoles. The collected vacuoles were resuspended in 20 mL of Buffer A and homogenized again with loosely fitting homogenizer, and then layered on top by 10 mL of Buffer B (10 mM MES-Tris, pH 6.9, 0.5 mM MgCl_2_, 8% Ficoll), and centrifuged as above. The intact vacuoles were collected and then converted to vacuolar membrane vesicles by diluting and homogenizing them with Buffer C (10 mM MES-Tris, pH6.9, 5 mM MgCl_2_, 25 mM KCl) using a tightly fitting Dounce Homogenizer. The vesicles were recovered as the pellet by centrifugation at 37,000 × *g* for 30 min at 4 °C.

The vacuolar membrane vesicles prepared as described above enriched a vacuolar membrane protein Vph1, whereas the vesicles contained no significant amount of cytosolic-, ER-, or mitochondrial-resident proteins, Pgk1, Dpm1, or Por1, respectively (Supplementary Fig. S6).

### ATP-dependent uptake of arginine by vacuolar membrane vesicles

The vesicles were incubated in the assay buffer (25 mM MES-Tris, pH 6.9, 5 mM MgCl_2_, and 25 mM KCl) at 25 °C for 1 min with or without 0.5 mM ATP. The reaction was initiated by the addition of [U-^14^C]arginine (final 100 μM; 10.14 GBq/mmol, Perkin-Elmer) at 0 min. After 0.5, 2, and 4 min, a 100 µL aliquot (40 μg of protein) of the reaction mixture was taken and diluted with 5 mL of ice-cold assay buffer to stop the reaction. Vesicles were recovered by suction on a cellulose acetate membrane filter (0.45 μm; ADVANTEC) and washed immediately with 5 mL of ice-cold assay buffer. The radioactivity remaining in the vesicles was determined using a liquid scintillation counter. l-[^14^C] labeled amino acids were purchased from American Radiolabeled Chemicals Inc. (St Louis, MO, USA), GE Healthcare (Buckinghamshire, UK), or Perkin Elmer Inc. (Waltham, MA, USA).

### Arginine uptake driven by an artificially imposed histidine gradient

Purification of vacuolar membrane vesicles was performed as described above with a modification using Buffer C containing 10 mM histidine to disrupt the intact vacuoles in the final step of isolation. The vacuolar membrane vesicles (40 μg of protein) were quickly diluted 50-fold at 15 °C with the assay buffer containing 50 μM [^14^C]arginine. For the control without a histidine gradient, the vesicles were diluted with the assay buffer containing 50 μM [^14^C]arginine and 10 mM histidine. To assess the inhibition of arginine-histidine exchange activity by the addition of non-labeled amino acids, assays were carried out in the presence of 500 μM (Fig. 4) or 1 mM (Supplementary Fig. S3) non-labeled amino acids.

### Western blotting analysis and antibodies

Total cell lysate or vacuolar membrane vesicles were subjected to 10% SDS-PAGE and analyzed by Western blotting using anti-HA (3F10, Roche), anti-Vph1 (10D7, Molecular Probes), and anti-Pgk1 (22C5, Molecular Probes) antibodies.

### Fluorescence microscopy

Fluorescence microscopy was carried out using Olympus IX71 fluorescence microscope. Vacuolar membranes were stained with fluorescent dye FM4-64 as described^22^.

## Supplementary information

Supplementary information

Supplementary information
