# Supplementary material for: A PQ-loop protein Ypq2 is involved in the exchange of arginine and histidine across the vacuolar membrane of Saccharomyces cerevisiae
Source: Sci Rep. 2019 Oct 21;9:15018. doi: 10.1038/s41598-019-51531-z (PMC6803629; doi:10.1038/s41598-019-51531-z)
Supplement: Supplementary file 1 — Supplementary information [file 41598_2019_51531_MOESM1_ESM.pdf]

A PQ-loop protein Ypq2 is involved in the exchange of arginine and histidine across the vacuolar membrane of *Saccharomyces cerevisiae*

Miyuki Kawano-Kawada, Kunio Manabe, Haruka Ichimura, Takumi Kimura, Yuki Harada, Koichi Ikeda, Shiho Tanaka, Yoshimi Kakinuma, Takayuki Sekito

Supplementary Figure S1

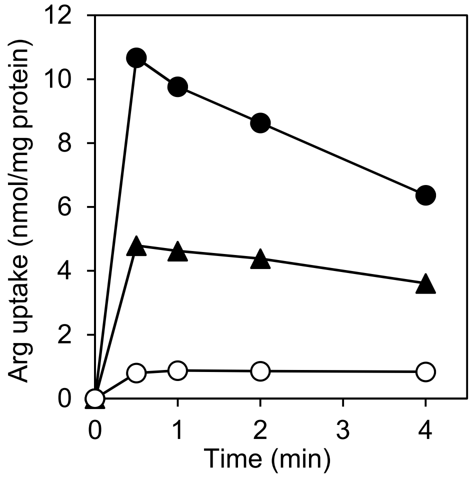

Supplementary Fig. S1. Effect of the levels of histidine gradient on the exchange activity.

Vacuolar membrane vesicles were pre-incubated with 10 mM histidine at 25˚C for 6 min, and the reaction was started by dilution of the mixture 50-fold with the assay buffer containing 53 μM [^{14}C]arginine along with 0.2 mM histidine (black circles), 1 mM histidine (black triangles), or 10 mM histidine (white circles). The reactions were carried out at 15 ºC. Results are mean of two independent experiments.

Supplementary Figure S2

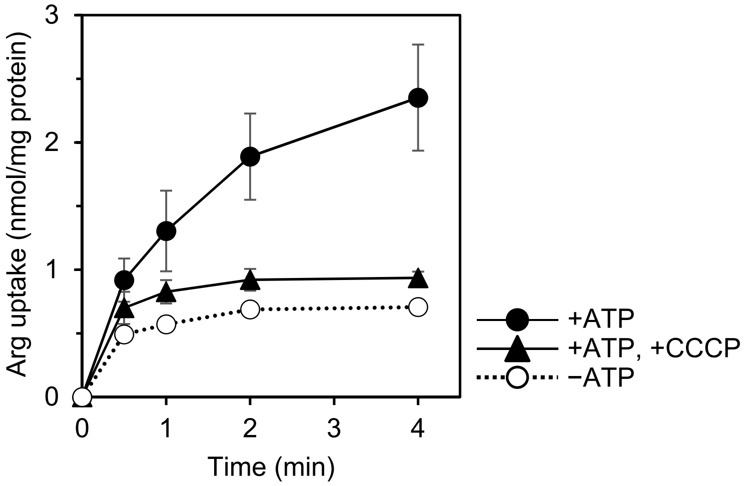

Supplementary Fig. S2. ATP-dependent uptake of arginine by vesicles of *ypq1*Δ*ypq2*Δ cells expressing *YPQ1.*

Vacuolar membrane vesicles isolated from the *ypq1*Δ*ypq2*Δ cells harboring pYPQ1-HA[3] was preincubated with (solid lines) or without (dotted lines) 0.5 mM ATP for 1 min. Before initiating the reaction, vesicles were incubated with 5 μM of CCCP at 25ºC for 3 min (black triangles). As controls, vesicles were incubated with solvent (black and white circles). The reaction was initiated by the addition of 50 μM [^{14}C]arginine at 0 min. Results are mean ± SD from three independent experiments.

Supplementary Figure S3

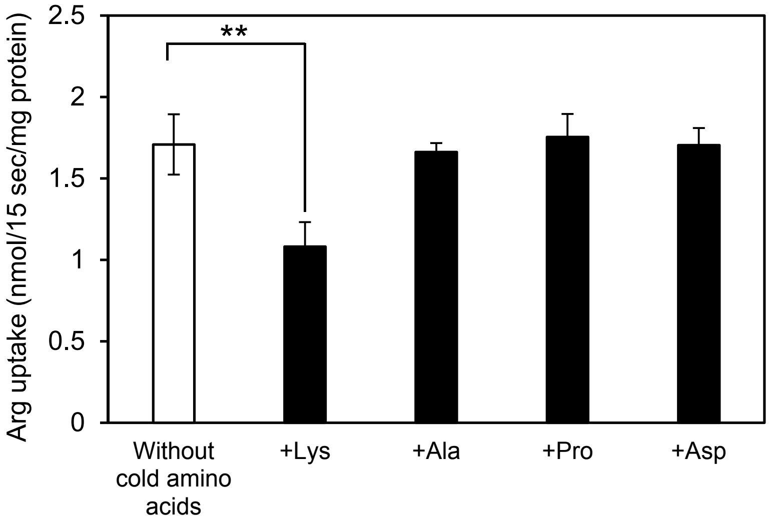

Supplementary Fig. S3. Inhibition of *YPQ2*-dependent arginine /histidine exchange activity by the addition of non-labeled amino acids.

Vacuolar membrane vesicles containing 10 mM histidine prepared from wild-type cells were incubated with 50 μM [^{14}C]arginine in the presence of 1 mM non-labeled amino acids as indicated (black bars). The initial rates of arginine uptake were determined at 15 sec. The control (without the addition of non-labeled amino acids) is shown as a white bar. Results are indicated as mean ± SD from three independent experiments. Significant differences from the control are indicated by asterisks (** p<0.01, Student's t-test).

Supplementary Figure S4

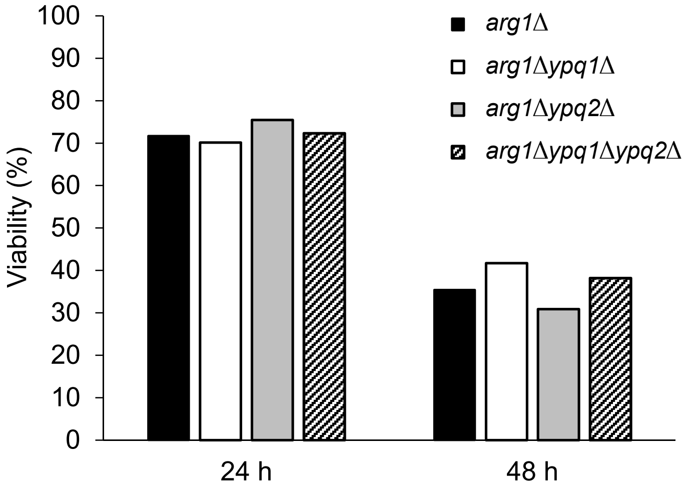

Supplementary Fig. S4. Effect of *YPQ*-disruption on the viability of cells grown in arginine-deprived medium.

Cells were grown at 30ºC until OD_{660} reached to 1.0 in the synthetic dextrose (SD) medium (0.17% yeast nitrogen base without amino acids and ammonium sulfate, 0.5% ammonium sulfate, and 2% glucose) supplemented with amino acids (SC medium). The cells washed once with SC medium without arginine (SC-Arg) were transferred to fresh SC-Arg medium at OD_{660}=1.0, and then further cultured at 30ºC. At indicated time, aliquots of cells were stained with phloxine B (4 μg/mL), which can not be excluded from the dead cells, and observed by fluorescence microscopy. The viable cells of *arg1*Δ (black bars), *arg1*Δ*ypq1*Δ (white bars), *arg1*Δ*ypq2*Δ (gray bars), and *arg1*Δ*ypq1*Δ*ypq2*Δ (hatched bars) were counted, and viability was calculated from the ratio of living cells to total cells. The average from two independent experiments is shown. All strains used carried pRS316 plasmid to complement *ura3* auxotrophy.

Supplementary Figure S5

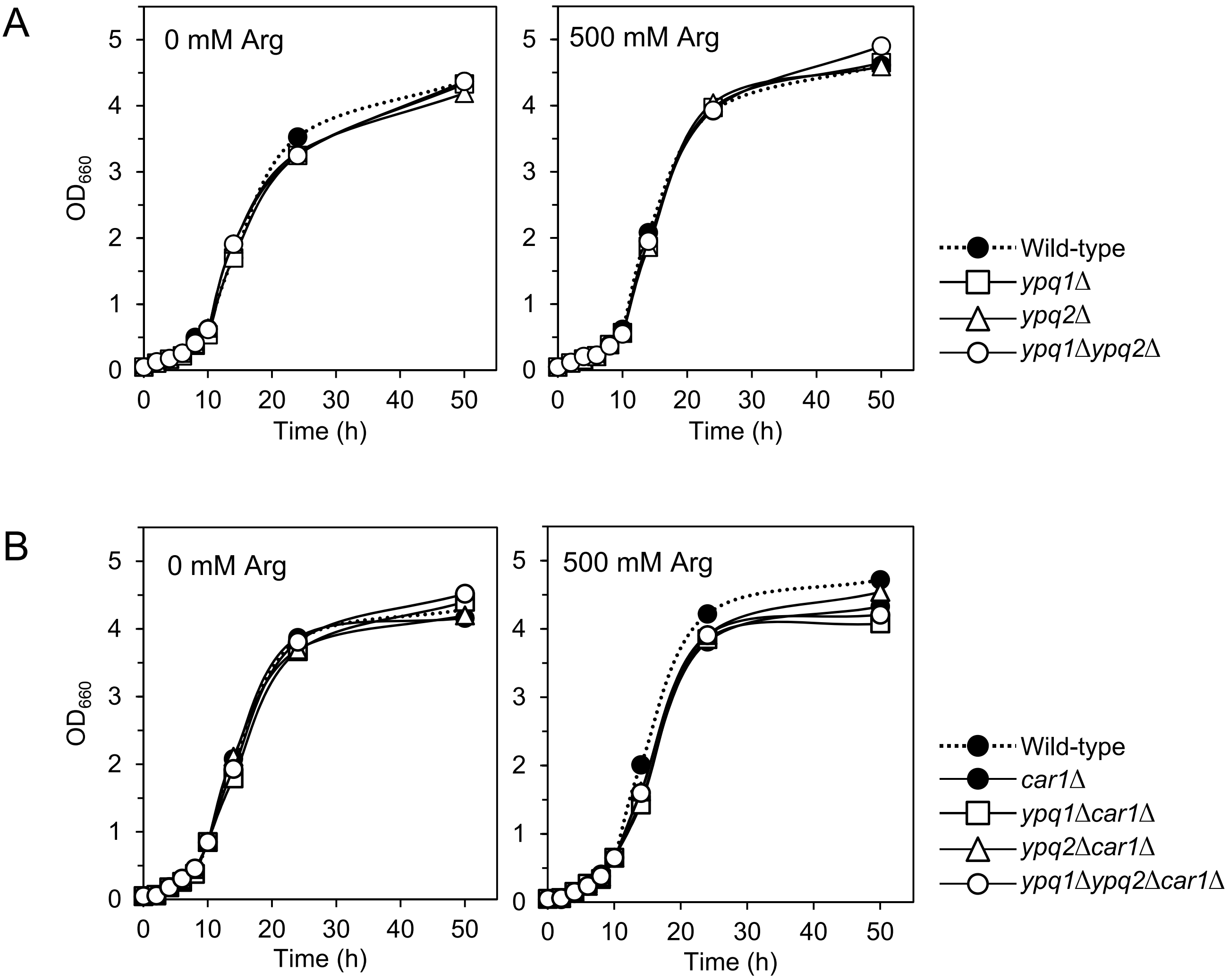

Supplementary Fig. S5. Effect of *YPQ*-disruption on the growth in medium containing high-dose arginine.

Cells grown at 30ºC until OD_{660} =1.0 in SD medium were diluted to OD_{660}=0.05 in 4 mL of SD medium supplemented with or without 500 mM arginine, and then further cultured at 30ºC. The growth of cells was monitored by measuring OD_{660}. All strains used carried pRS316 plasmid to complement *ura3* auxotrophy. Symbols: ● with dotted line, wild type; ● with solid line, *car1*Δ; □, *ypq1*Δ (A) or *ypq1*Δ*car1*Δ (B); △, *ypq2*Δ (A) or *ypq2*Δ*car1*Δ (B); ○, *ypq1*Δ*ypq2*Δ (A) or *ypq1*Δ*ypq2*Δ*car1*Δ (B).

Supplementary Figure S6

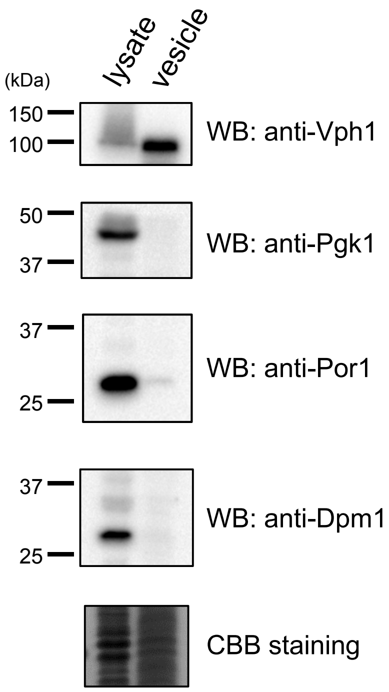

Supplementary Fig. S6. Western blotting analysis of total cell lysates and vacuolar membrane vesicles.

Twenty μg protein of cell lysates or isolated vacuolar membrane vesicles prepared from wild-type cells were subjected to 10% SDS-PAGE followed by Western blotting analysis using anti-Vph1, anti-Pgk1, anti-Por1 (16G9, Molecular Probes), or anti-Dpm1 (5C5, Molecular Probes) antibodies. CBB staining panel is indicated as a loading control.
