# Supplementary material for: A PQ-loop protein Ypq2 is involved in the exchange of arginine and histidine across the vacuolar membrane of Saccharomyces cerevisiae
Source: Sci Rep. 2019 Oct 21;9:15018. doi: 10.1038/s41598-019-51531-z (PMC6803629; doi:10.1038/s41598-019-51531-z)
Supplement: Supplementary file 2 — Supplementary information [file 41598_2019_51531_MOESM2_ESM.docx]

A PQ-loop protein Ypq2 is involved in the exchange of arginine and histidine across the vacuolar membrane of *Saccharomyces cerevisiae*

Miyuki Kawano-Kawada, Kunio Manabe, Haruka Ichimura, Takumi Kimura, Yuki Harada, Koichi Ikeda, Shiho Tanaka, Yoshimi Kakinuma, Takayuki Sekito*

Supplementary Table S1. Yeast strains used in this study.

| Strain | Genotype | Source |
| --- | --- | --- |
| X2180-1B | *MATα SUC2 mal mel gal2 CUP1* | Yeast Genetic stock Center |
| STY3807  STY5060 | X2180-1B *ura3*∆*::loxP*  STY3807 *ypq1*∆*::hphMX* | ^1)^  ^2)^ |
| STY5033 | STY3807 *ypq2*∆*:: kanMX* | This study |
| STY5073 | STY3807 *ypq3*∆*::loxP-kanMX-loxP* | ^3)^ |
| STY5071  STY3827  STY4969  STY6694  STY6699  STY6709  STY3961  STY6695  STY6698  STY6697 | STY3807 *ypq1*∆*::hphMX ypq2*∆*:natMX*  STY3807 *avt1*Δ::*loxP*  STY3807 *arg1*∆*:: kanMX*  STY3807 *arg1*∆*:: kanMX ypq1*∆*::hphMX*  STY3807 *arg1*∆*:: kanMX ypq2*∆*::natMX*  STY3807 *arg1*∆*:: kanMX ypq1*∆*::hphMX ypq2*∆*::natMX*  STY3807 *car1*∆*:: kanMX*  STY3807 *car1*∆*:: kanMX ypq1*∆*::hphMX*  STY3807 *car1*∆*:: kanMX ypq2*∆*::natMX*  STY3807 *car1*∆*:: kanMX ypq1*∆*::hphMX ypq2*∆*::natMX* | This study  ^4)^  This study  This study  This study  This study  This study  This study  This study  This study |
|  |  |  |

^1)^ Sekito, T., *et al*., *Biosci. Biotechnol. Biochem.* **78**, 969-975 (2014)

^2)^ Sekito, T., *et al*., *Biosci. Biotechnol. Biochem*. **78**, 1199-1202 (2014)

^3)^ Manabe, K., *et al*., *Biosci. Biotechnol. Biochem.* **80**, 1125-1130 (2016)

^4)^ Tone, J. *et al*. *Biosci. Biotechnol. Biochem*. **79**, 782-789 (2015)

Supplementary Table S2. Primers used in this study

| Primer | Sequence |
| --- | --- |
| 1F | 5’-GTATGGGAATACTATAGATCTAAG-3’ |
| 2R | 5’-CAAGCTCTTGAAACCTCGAGC-3’ |
| 3F | 5’-CTGGTGAAACGCAAACAGGATCCTAGTTTTCCCTACGGAG-3’ |
| 4R | 5’-CTCCGTAGGGAAAACTAGGATCCTGTTTGCGTTTCACCAG-3’ |
| 5F | 5’-GGTAGATCTATGTCGTGCTCAAACGGCATC-3’ |
| 6R | 5’-CAAGCTCTTGAAACCTCGAGC-3’ |
| 7F | 5’-CTCTGTTATTTCATTGTTTGCACAAATAATTGAGACTTATCG-3’ |
| 8R | 5’-CGATAAGTCTCAATTATTTGTGCAAACAATGAAATAACAGAG-3’ |
| 9F | 5’-CGTAGGCGCACGTATCGCACAACTAATCAAGAATTAC-3’ |
| 10R | 5’-GTAATTCTTGATTAGTTGTGCGATACGTGCGCCTACG-3’ |
